# Arachidonic acid as a potentially critical nutrient for vegetarians and vegans – position paper of the Research Institute for Plant-based Nutrition (IFPE)

**DOI:** 10.1186/s12944-025-02645-z

**Published:** 2025-07-19

**Authors:** Stine Weder, Sandra Müller, Christine Dawczynski, Markus Keller

**Affiliations:** 1 Research Institute for Plant-Based Nutrition (IFPE), Biebertal, 35444 Germany; 2 Junior Research Group Nutritional Concepts, Institute of Nutritional Sciences, Friedrich Schiller University Jena, Jena, 07743 Germany

**Keywords:** Arachidonic acid, LC-PUFAs, Vegetarian, Vegan, N-6 fatty acid, Pregnancy, Breastfeeding, Infancy, Childhood

## Abstract

The long-chain polyunsaturated fatty acid arachidonic acid (ARA, 20:4n-6) is virtually non-existent in plant foods. Concerns have therefore been raised that a predominantly plant-based diet, i.e., a vegetarian or vegan diet, could lead to ARA deficiency. ARA is discussed as conditionally essential, particularly in early infancy and childhood. Therefore, the authors of this position paper provide an overview of the state of scientific research on ARA for Western countries, with a special focus on risk groups, e.g., pregnant and breastfeeding women, infants, and children. We discuss whether ARA is potentially essential in critical life stages and whether a plant-based diet is appropriate in relation to ARA. Preliminary recommendations are derived on the basis of the available literature: healthy adults and pregnant and lactating women are able to synthetize ARA from linoleic acid (LA, 18:2n-6) and, therefore, do not require ARA supplementation, even with plant-based diets. Owing to the importance of ARA in early childhood, infants should be breastfed or given ARA-containing formula. According to current data, it can be assumed that infants, children, and adolescents are also capable of producing sufficient amounts of ARA regardless of their diet. However, these data are sparse. Furthermore, owing to the lack of reference ranges, there are no suitable test methods for diagnosing deficiency. All in all, owing to insufficient data, these recommendations are preliminary, and further studies with risk groups are urgently needed.

**Supplementary Information:**

The online version contains supplementary material available at 10.1186/s12944-025-02645-z.

## Introduction

Arachidonic acid (ARA, 20:4n−6) is an n-6 20-carbon fatty acid (FA) and is a long-chain polyunsaturated FA (LC-PUFA). The prevailing view is that the human body can synthetize ARA in sufficient quantities by itself. Recently, however, ARA has been considered (semi)essential, especially in infants and possibly in plant-based diets [1–4]. ARA is found mainly in animal foods, leading to the assumption that vegans consume no ARA and that vegetarians consume less ARA than omnivores do. Furthermore, the body can synthesize ARA from the essential FA linoleic acid (LA, 18:2n−6). However, there are phases in life, such as early childhood, when this biosynthesis may not be sufficient. In addition, genetic factors have an inhibitory influence on ARA synthesis and can be associated with lower ARA levels. As ARA is considered essential in early childhood, the question has recently arisen in the (scientific) discussion, whether a plant-based diet, particularly a vegan diet, is sufficient to cover the physiological requirements for ARA [1, 5]?

Therefore, this position paper aims to

- present the current state of research regarding the intake and status of vegetarians and vegans with ARA, with a particular focus on critical phases of life around early childhood (pregnancy, breastfeeding, infancy) as well as childhood and adolescence.
- derive preliminary/initial recommendations for dietary and/or supplemental intake of ARA for vegetarians and vegans.

## Methods

### Biosynthesis, metabolism, and physiological functions in the human body

**Fig. 1:**
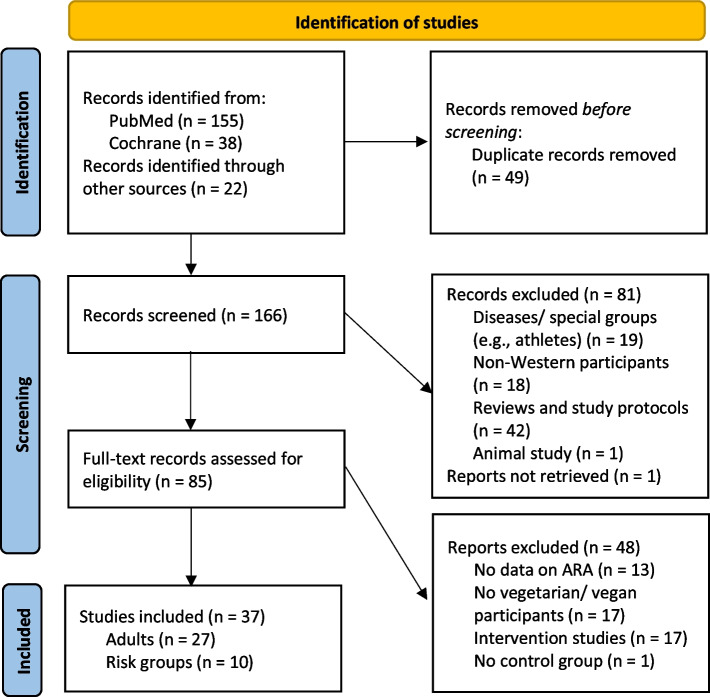
Prisma flow diagram [created according to [137]]. This work is licensed under CC BY 4.0. To view a copy of this license, visit https://creativecommons.org/licenses/by/4.0/

PubMed and Cochrane were searched for studies published up to the 12th of March 2025. We used keywords in search strings containing different terms for arachidonic acid (20:4n-6 OR long-chain polyunsaturated fatty acids OR PUFA OR 20:4omega6 OR or fatty acids) in combination with vegetarian diets (vegan OR vegetarian OR plant-based) and terms for risk groups (breastfeeding OR lactating OR breast milk OR pregnant OR children OR infant OR toddler OR adolescent). We then searched the reference lists of the studies found for further studies. In addition, statements regarding ARA from medical professional and nutritional societies were screened. We found 166 records with humans that were available in English or German. After the elimination of duplicates, reviews, and study protocols, as well as records focusing on diseases, we focused on studies with people in Western societies. We defined Western societies or Western diets according to Clemente-Suárez et al. (2023) as a diet that is “a modern dietary pattern that is characterized by high intakes of processed and refined foods, red and processed meats, added sugars, and saturated and trans fats and low intakes of fruits, vegetables, whole grains, and nuts” [6], which predominates in Western societies like Europe, North America, and Oceania, in contrast to more traditional, i.e. unprocessed, diets practiced e.g. in Asia or Africa [6, 7]. According to Clemente-Suárez et al. the “Western diet countries” include “Iceland, Switzerland, the United States, Australia, Sweden, Hungary, France, Austria, Germany, Denmark, the Czech Republic, the Netherlands, Spain, Belgium, Finland, and New Zealand”, a definition based on a calculation based on official FAO data [6]. We excluded studies from non-Western countries because the respective populations not only differ from non-Western societies in their dietary habits, but also genetically. Moreover, studies without data on ARA, or vegetarian/vegan participants or without control group as well as intervention studies were excluded. We finally identified 27 studies reporting the ARA intake and/or ARA status of vegetarian/vegan adults. Another three studies included pregnant women, four studies included breastfeeding women, and only three studies included children or adolescents on a plant-based diet (Fig. 1).

## Physiological background

### Biosynthesis, metabolism, and physiological functions in the human body

**Fig. 2:**
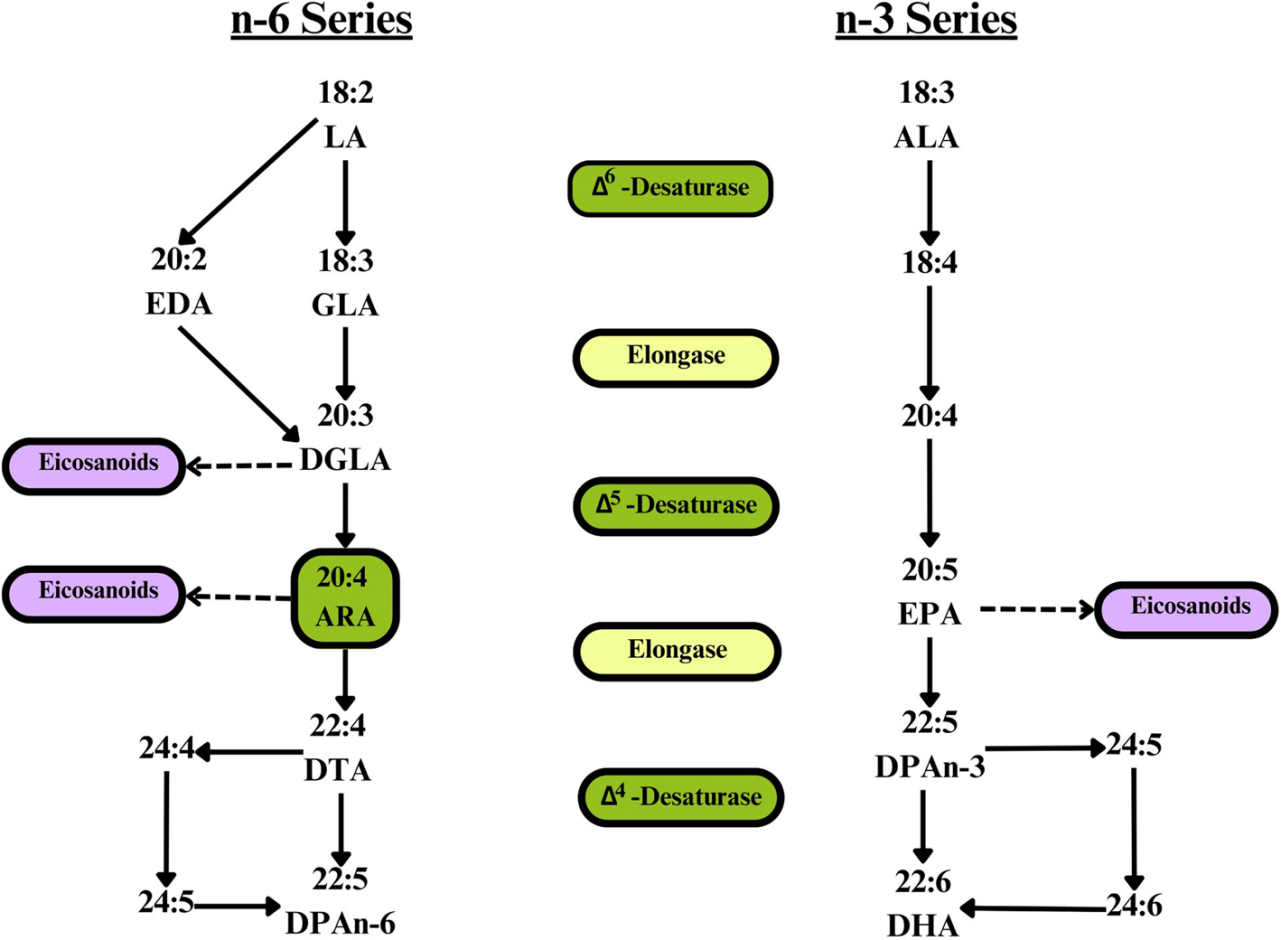
Metabolic pathways of the n-6 and n-3 FA series [35, 41]. Abbreviations: ALA, α-linolenic acid; ARA, arachidonic acid; DHA, docosahexaenoic acid; DGLA, dihomo-γ-linolenic acid; DPA, docosapentaenoic acid; DTA, docosatetraenoic acid (adrenic acid); EPA, eicosapentaenoic acid; EDA, eicosadienoic acid; FA, fatty acid; GLA, γ-linolenic acid; LA: linoleic acid

In addition to the dietary intake of ARA, the LC-PUFA is formed by desaturation and elongation from the essential FA linoleic acid (LA, 18:2n-6). Further biosynthesis steps produce docosapentaenoic acid (DPAn-6, 22:5n-6) (Fig. 2). This pathway competes with the biosynthesis of n-3 FAs eicosapentaenoic acid (EPA, 20:5n-3), docosapentaenoic acid (DPAn-3, 22:5n-3), and docosahexaenoic acid (DHA, 22:6n-3) from the essential FA α-linolenic acid (ALA, 18:3n-3) for the corresponding enzymes [3]. For example, high amounts of dietary ALA could competitively inhibit desaturation and elongation of LA and therefore the conversion of LA into ARA [4]. However, LA intake usually exceeds that of ALA many times [8]. PUFA synthesis thus depends not only on several factors, such as substrate availability/competition and enzyme activity (e.g., Δ−5 and Δ−6 desaturases) but also on the availability of specific nutrients (e.g., zinc, magnesium, and calcium) and genetics [9, 10]. However, Dawczynski et al. did not find any influence of micronutrient status on LA conversion [11]. The conversion rate from LA to other n-6 PUFAs is estimated in stable isotope studies to be 1–2.2%, and the conversion rate is only 0.2–0.6% for LA to ARA in healthy individuals [12–14]. In lactating women, 1.1–1.2% of human milk ARA originates from the conversion of LA [15, 16]. This low rate may further decrease with increasing age [17]. The conversion rate in women of childbearing age may be higher than that in men, as is the case for the conversion of ALA to EPA/DHA [10]. Nutritionally important n-6 and n-3 PUFAs are shown in the supplementary material (see Supplementary Table 1, Additional File 1).

ARA is the most abundant LC-PUFA as part of phospholipids (PL) in cell membranes. It is essential for cell integrity, membrane properties (i.e., permeability, flexibility, fluidity), order, and the vascular system and is important for the immune system, signal transduction, and gene transcription [3, 4, 18, 19]. Furthermore, ARA, in addition to other LC-PUFAs, is the primary precursor of eicosanoids (Fig. 2) [3, 4]. Eicosanoids occur in almost every tissue of the body and thus have a variety of critical and specific functions, including cardiovascular, pulmonary, renal, reproductive, and secretory functions, and they are vital for bone turnover and the release of hormones, which are important for cell proliferation and growth. ARA is also important for muscle repair and growth; therefore, it is interesting for athletes and might have protective potential against cancer [3, 4, 19, 20].

Furthermore, eicosanoids are important for the immune system, i.e., as both mediators and regulators of inflammation. ARA is a substrate for inflammation-promoting eicosanoids such as LTB_4_ (responsible for inflammation symptoms) [21]. In addition, an eicosanoid formed from ARA is PGE_2,_ which has both pro-inflammatory functions (induces fever and increases vasodilatation) and anti-inflammatory functions (inhibits the production of tumor necrosis factor [TNF] and interleukin [IL]−1) [3], although the proinflammatory response seems to predominate [22].

### ARA in infant development

The FA metabolism and needs of infants differ uniquely from those of adults. Therefore, direct extrapolation of the metabolism and functions of these FAs in adults to infants is not possible [23]. Thus, the particularities and functions of ARA in the development of infants are explained here.

Unlike other FAs, such as DHA, ARA transfer via the placenta to the fetus in utero does not appear to be related to maternal ARA status or maternal ARA intake [23]. Furthermore, after birth, the median ARA content in maternal milk is stable at approximately 0.5% (0.24–1.0%) of milk FAs or 140 mg per day throughout exclusive/full breastfeeding [23–27] because it mainly (~ 90%) originates from maternal internal storage. Thus, dietary intake seems to have only a minimal influence on ARA breast milk concentrations either during pregnancy or postpartum [15]. In contrast, Weseler et al. conducted a small (*n* = 33) supplemental, double-blind, controlled trial showing a significant and dose-dependent increase in ARA concentrations in human milk dependent on supplement intake (i.e., from 0.47% to 0.56% with 400 mg ARA/d in addition to DHA, EPA, and other n-3 FAs for eight weeks, p ≤ 0.001), whereas in the control, ARA concentrations decreased [28]. However, the increase in the ARA levels was only moderate.

The accumulation of ARA and its elongation products in fetal and infant organs, tissues, and membranes is important for early child development, e.g., for brain growth during gestation and early infancy, as well as the immune system [3, 23, 29]. This finding supports, together with the fixed concentration of ARA in human milk, “the concept of the essentiality of ARA” in this sensitive growth phase [3]. Furthermore, ARA and other LC-PUFAs are incorporated into the developing brain approximately ten times more efficiently than ALA and LA are [4].

Stable isotope studies suggest that ARA can be synthesized from LA as early as the 26th week of pregnancy. However, they reported that the conversion rate of LA to ARA decreases with increasing gestational age [30]. In addition, after birth, infants can synthesize ARA, but endogenous synthesis activity seems rather low, and ARA in the blood shortly decreases after birth [31, 32]. The endogenous ARA synthesis rate was reported to be 67% at 1 month, 36% at 3 months, and 29% in 7-month-old preterm-born infants [33]. This decline after birth demonstrates the likely insufficient biosynthetic capability to meet the infant’s demand [31, 34]. In this context, it is not yet clear whether the synthesis rate is decreasing because demand is decreasing. This is confirmed by studies showing a severe decrease of up to 40% in ARA shortly after birth when individuals consume formulas without ARA (especially in preterm infants) [3, 33].

With the introduction of complementary feeding, the ingested volume of breast milk, an important source of ARA (and DHA), decreases. The exclusive consumption of foods without ARA (and DHA) might lead to the depletion of these LC-PUFAs in the body [18]. As weaning foods generally contain low amounts of fat and, thus, ARA and DHA [3], paying particular attention to complementary foods containing ARA and DHA [18] is crucial.

In conclusion, growing infants need ARA (in human milk or infant formula) to maintain their ARA blood levels, which is sufficient to meet their metabolic needs. The target value is the concentration achieved by breastfed infants [3, 23]. Whether ARA intake is too low when weaning foods are introduced has not been conclusively clarified.

### Consequences of ARA deficiency/long-term low ARA intake

#### In general

In many cases, distinguishing the specific effects of ARA deficiency separately from those related to deficiency of other n-6 FAs, especially LA, is difficult [3]. Indeed, many of the biological functions associated with LA and ALA are induced by their metabolites, including dihomo-γ-linolenic acid (DGLA), ARA, EPA, DPA, and DHA [35]. Since essential FAs are necessary for the normal functioning of all tissues, a LA deficiency results in a variety of symptoms, including reduced growth rates, scaly dermatitis, infertility, depressed inflammatory responses, kidney and liver abnormalities, decreased capillary resistance, increased fragility of erythrocytes, and reduced contraction of myocardial tissue [4]. Nevertheless, owing to the high availability of LA and ALA in the diet, deficiencies in humans are rare [4, 36, 37]. A deficiency of the LC-PUFAs in healthy adults, especially ARA, EPA or DHA, has also never been observed, as the FAs mentioned are supplied with food or, despite the low synthesis rates, are formed in sufficient quantities over the long term. However, a deficiency can occur during the perinatal period (see below) [38].

In addition, there are genetic variants in the gene cluster of FA desaturase enzymes that modify their activity to convert precursors into LC-PUFAs and, thus, the composition of blood and tissue lipids. A strong influence of genotype was found for ARA, as almost 30% of the variation in serum phospholipid levels could be predicted by it. This effect size is much larger than the variation achieved in dietary intervention studies. In contrast, the effects on DHA and other n-3 PUFAs are small and, in most studies, non-significant [35]. In Europe, 25–50% (Germany: 28%) of the population appear to be “slow converters” (haplotype A), leading to 24% lower DHA and 43% lower ARA levels than those of haplotype D [35, 39, 40]. This finding is in line with another study revealing that 25% of the European population possesses the “slowest converter” haplotype (here D/D), resulting in a 31% lower conversion of ARA to LA than the “fastest converter” haplotype (here I/I) [41]. Nevertheless, the conversion from the LA to the ARA also appears to be sufficient in slow converters. A lower ARA concentration might even be advantageous regarding inflammatory diseases [39] (see discussion).

Chowdhury et al. (2014) conducted a meta-analysis of prospective cohort studies and randomized controlled trials (RCTs). The meta-analysis revealed that higher ARA rates were associated with decreased cardiovascular risk (*n* = 10, RR for coronary outcomes 0.83 [95%CI 0.74–0.92] comparing top vs. bottom thirds). However, the results of RCTs with n-3 and n-6 PUFA supplementation suggested that this did not significantly reduce the risk for coronary outcomes [42]. The authors of another review with four RCTs reported “no statistically significant effects of either increased or decreased omega-6 intake on CVD risk factors” [43]. However, they did not report the individual effects of ARA. A recent meta-analysis of 21 cohorts and 11 case‒control studies revealed an inverse association for LA and CHD risk (RR: 0.85 95% [CI 0.71–1.00]), but not for ARA risk [44].

#### Infants (and children)

Independent of LA, adequate intake of ARA seems important for growth and the immune system, especially in fetal and infant development [4]. Thus, ARA deficiency in infants leads to adverse effects on growth (especially in premature infants) [3, 23]. It is also associated with a higher incidence of childhood stunting and infant mortality [45] or slower growth rates [46]. As precursors for many eicosanoids, preterm infants suffer from medical issues linked to “vascular (e.g., retinopathy of prematurity, periventricular hemorrhage, necrotizing enterocolitis) and immune (general infection, necrotizing enterocolitis) functions” [47]. Moreover, consuming formula with DHA but without ARA led to a higher risk of cardiovascular and cerebrovascular morbidity and mortality [23, 31].

Variations in the desaturase gene cluster, resulting in low activity of the ∆−5 and ∆−6 desaturating enzymes and thus low ARA concentrations, are associated with eczema, asthma, allergic rhinitis, and lower cognitive outcomes in infants [23, 40, 48]. Conversely, the usual provision of ARA during breastfeeding appears to be associated with a lower incidence of asthma and better cognitive development, at least in infants with a low intrinsic synthesis of ARA [23]. Both maternal and child genotypes influence cord venous plasma FA, with a greater effect of the child genotype on ARA [49]. Consequently, infants with low desaturase activity, as well as infants with mothers with low desaturase activity, may require higher amounts of ARA to maintain an optimal status. However, the content of ARA in breastmilk may have compensated for decreased desaturase activity in three-month-old breastfed infants [48, 50].

The German Nutrition Society (Deutsche Gesellschaft für Ernährung, DGE) classifies DHA and ARA as “conditionally essential nutrients”, which should also be supplied with infant formulas [38]. Le et al. (2009) generally question the essentiality of LA (and ALA) and instead advocate the essentiality of ARA (and DHA/EPA) [36].

### Consequences of excess/long-term high ARA intake and interaction with other FA

There are two ways to influence ARA concentrations in the body: self-synthesis from LA and the intake of preformed LC-PUFAs from food. An excess of ARA due to self-synthesis seems unlikely, as a systematic review of 36 articles revealed that increasing LA in the diet (as much as 551% from baseline) did not increase plasma/serum and red blood cell (RBC) levels of ARA in adults on a typical Western diet. This may not be a result of tissue saturation of ARA but of the limited conversion by the delta-6 desaturase [51]. However, in one intervention study with patients with rheumatoid arthritis (RA), a significant increase in LA and ARA levels was detected due to increased LA intake via sunflower oil (4.7 g LA/d) within 10 weeks (+ 4.3% of FA methyl esters in RBC lipids) [52].

In contrast, dietary GLA and ARA increase ARA levels in plasma/serum PL [51]. This finding was confirmed by another review of nine studies that investigated the effects of ARA supplementation: all doses of ARA supplements significantly increased ARA levels in a dose-dependent manner (82–3600 mg/kg, *r* = 0.87), regardless of whether DHA or EPA was supplemented. This was true even with doses comparable to or lower than the average dietary ARA intake range (82–120 mg/d for 3–4 weeks). Furthermore, LA, but not the DHA/EPA ratio, was decreased by ARA supplementation, whereas DHA/EPA supplementation decreased the ARA composition [53]. Similarly, a systematic review of 14 RCTs investigated (potentially positive) the effects of increased intake of ARA (40–2000 mg, 1–12 weeks) on FA status and health outcomes in humans. They reported no negative effects of high ARA dosages up to 1500 mg per day on blood lipids, platelet aggregation/blood clotting, immune function, inflammation, or urinary excretion of ARA metabolites, but EPA concentrations were decreased in several studies. Furthermore, there was only a small advantage (e.g., body composition [increased lean body mass, reduced fat mass], muscle function, and physical performance [e.g., improved peak power]) of supplementing ARA in healthy adults, whereas elderly people might benefit from it because of the impact of ARA on cognitive and muscle function. However, studies in many fields are lacking, and the maximum intervention time was twelve weeks. Thus, the effects of increased ARA intake must be investigated further [20]. Moreover, caution is advised regarding ARA supplementation in patients with inflammatory diseases [54].

Other FAs (mainly LA, EPA, and DHA) in addition to ARA also influence the ARA blood status. The ability to incorporate or store FAs in the plasma PL/body appears to be in the following order: DHA/EPA > ARA > LA, potentially because of enzyme–substrate specificities. Thus, supplementation with EPA and DHA dose-dependently decreases ARA concentrations in plasma/serum [53, 55]. However, the current state of research is inconsistent with the question of at which dosage this effect starts. For example, Schuchardt et al. investigated the effect of ~ 1 g DHA/d on PUFA concentrations in the plasma and RBCs of 12 healthy men. They reported a time-dependent decrease in ARA levels, especially in plasma (−24%), compared with those in RBCs (−16%) after 12 weeks of supplementation. The authors explained the effect of the displacement of ARA by DHA and decreased desaturase activity, which resulted from the lower conversion of LA to ARA [56]. Although these findings are in line with other studies [57–63], the amounts of DHA used in these studies were mostly, but not always [57, 60, 62, 63], relatively high (> 1 g/d). In contrast, other studies with lower doses of DHA, which correspond approximately to the amount recommended [59, 60, 64] by national and international bodies (250–500 mg EPA + DHA/d, [65]) or ≤ 1 g/d [59, 64, 66], revealed no, neglectable or nonsignificant effects on ARA levels. Even some studies with DHA concentrations ≥ 1 g/d reported no significant effects on ARA concentrations [64, 66, 67]. A DHA-rich oil (1.5 g DHA, 0.6 g EPA, 29 mg ARA) derived from *Schizochytrium* sp. increased ARA plasma concentrations in an RCT with 79 participants for 4 weeks, with no significant effect on RBC PL FA composition [68].

The study situation is therefore very inconclusive. However, in the range of the recommended daily EPA + DHA intake by national and international bodies (250–500 mg/d), ARA concentrations did not decrease or only moderately decreased. The clinical effects of moderately lower ARA levels are not known and could also have positive effects on inflammation in the body. Therefore, DHA supplementation *without* ARA appears to be safe only at the recommended doses of up to 250–500 mg EPA + DHA/d for people on a plant-based diet to avoid a decrease in ARA levels because of supplementation. This is in accordance with a supplementation RCT with 116 vegetarian, vegan, and omnivorous adults in Spain, where 250 mg DHA/d did not significantly change the ARA concentrations in serum FAs over 5 weeks in all diet groups [69]. Another study of the same working group with 49 vegetarians and 55 vegans revealed no significant differences in the ARA profiles (in %) of n-3-supplemented (*n* = 95) and non-supplemented (*n* = 9) subjects. Although the group sizes were not balanced and very diverse n-3 supplements were used, there was a significant difference in the DHA profile (in %) [70]. A previous study in which a high dose of DHA (0.94 g/d) was consumed daily for 8 weeks reported a significant decrease in mean plasma ARA levels (from 8.9 ± 0.2 to 8.0 ± 0.2 g/100 g FA, *p* < 0.001) at 114 vegetarians [71]. In contrast, high DHA intake without balanced amounts of ARA (ARA:DHA ≥ 1:1) in infants may result in low ARA levels in brain tissues, suboptimal neurodevelopment, and possibly negative effects on growth and immune development [23].

ARA is the major precursor for eicosanoids, and eicosanoid synthesis is regulated by the availability of ARA in PL. Thus, it was assumed that the more ARA is present, the greater the n-6 eicosanoid signal from the released PUFAs. An imbalance in eicosanoid production can lead to a series of inflammatory and autoimmune disorders, e.g., thrombosis, immune-inflammatory disease (i.e., arthritis, lupus nephritis), cancer, and psoriatic skin lesions. This concern about imbalanced eicosanoid production also exists for a high intake of LA relative to n-3 PUFAs, leading to different suggestions for an optimal n-6:n-3 ratio [4]. Currently, the DGE (still) recommends a dietary LA:ALA ratio of < 5:1 [72]. However, the realization that “not all n-6 FAs are bad”, i.e., proinflammatory, and “not all n-3 FAs are good”, i.e., anti-inflammatory, has led to louder calls to abandon the n-6:n-3 ratio and use, e.g., the n-3 index instead [73]. Indeed, it was shown that higher dietary intakes or blood status of LA relative to lower intakes/statuses are beneficial for cardiometabolic health and are associated with reduced inflammatory status in healthy adults [73]. Furthermore, ARA supplementation (240 or 720 mg/d), which increased ARA tissue concentrations in one study, did not stimulate an inflammatory response (related to cardiovascular, inflammatory, and allergic diseases) or altered its metabolites in healthy Japanese elderly individuals [55]. In contrast, lower EPA/DHA levels lead to a greater inflammatory status; thus, “the problem is not the presence of the n-6 FAs but the absence of the n-3 FAs” [73]. This finding is in line with a study that showed that an increase in EPA and DHA intake can attenuate a disturbed mood caused by changes in the brain due to high ARA intake [74]. However, it must be kept in mind that this may only apply to healthy (young) humans, as, e.g., in patients with RA, a higher LA intake (via sunflower oil) was associated with a significant increase in ARA concentrations and the production of proinflammatory mediators [52]. In general, everyone is at risk of undetected inflammation, and the prevalence of inflammatory diseases has risen in recent years (Germany: estimated 2.2–3.0% of adults with rheumatic diseases), especially with increasing age [75, 76]. Notably, vegetarian–vegan diets are associated with reduced systemic inflammation in healthy adults and improved clinical symptoms in RA patients [54, 77–81].

Overall, textbook knowledge contrasts (more or less) with more recent findings that healthy humans are not negatively influenced by an ARA intake of up to 1.5 g/d [20]. However, this might only be true for healthy adults and needs further research in individuals with, e.g., cardiovascular, inflammatory, and allergic diseases [55]. It is suggested that n-6 FA intake should not be limited, but one should rather balance ARA intake with EPA/DHA intake [10, 73]. In this context, supplementation with DHA+EPA *without* ARA up to 250 mg per day does not seem to negatively affect the ARA status.

## Recommended and actual intakes for ARA

### Recommended intakes for ARA for the general population

For Europe, the European Food Safety Authority (EFSA) does not classify ARA as an essential FA and thus proposes no dietary reference value (DRV) except for adequate intake (AI) for infants aged 0 to < 6 months. Additionally, no UL for any n-6 PUFA was detected [82, 83]. Other ARA intake recommendations in Europe are limited and exist only for infants (Table 1). For pregnant and breastfeeding women, recommendations refer only to DHA intake [84]. Although not proposing a DRV for infants, the DGE notes that DHA and ARA can be considered “conditionally essential nutrients that should also be supplied with infant formulas” [72]. In addition, (supplementation of) ARA in elderly individuals “has recently gained increased attention” [55]. This might be due to a more fragile PUFA status in elderly individuals (women), who might be more dependent on the exogenous supply of ARA due to lower ARA biosynthesis [17].

**Table 1:** European and international recommendations for ARA intake

Institution/Country (reference)	Age/Risk group	Recommendation
EFSA/Europe [83]	Infants 0- < 6 mo	140 mg/d (AI)
French Food Safety Agency/France [85]	Infants 0–6 mo	0.5% of total FAs
FAO of the UN and WHO [86]	Infants 0–6 mo	0.2–0.3 E% (0.4–0.6% of total FAs)
Health Council of the Netherlands/Netherlands [87]	Infants 0–5 mo	40 mg/kg bodyweight

*Abbreviations*: *EFSA* European Food Safety Authority, *E%* % of energy, *FAO* Food and Agriculture Organization, *mo* months of age, *WHO World Health Organization,* *y* years of age

### Actual intakes of ARA in the general population

#### Adults

The mean ARA intake in the German European Prospective Investigation into Cancer and Nutrition (EPIC) cohorts (Heidelberg/Potsdam) was 160/140 mg/d for women and 230/230 mg/d for men [88] and thus fits into the estimated ranges specified for developed countries (ranging from 101–351 mg/d [89] and 100–250 mg/d [53]). The mean LA intake was 10.9/11.6 g/d (women) and 14.3/18.6 g/d (men), and the mean ALA intake was 1.3/1.5 g/d (women) and 1.6/2.3 g/d (men). The mean proportion of total n-6 FAs in men and women was between 5.3% and 6.5% of energy (E%), and that of total n-3 FAs was between 0.7 E% and 0.9 E%. The dietary n-6:n-3 ratio of the average population in Germany is estimated to be 7.2–8.0 (women) and 7.7–8.6 (men) [88].

#### Risk groups in general

According to a systematic review, there are very few data (number of studies in brackets) in European countries on ARA intake in specific population groups, such as infants (1), toddlers (1), children (2), adolescents (8), elderly individuals (4), and pregnant (3)/lactating (1) women [84]. The rates of ARA intake reported in these studies are presented in Table 2 [84].

**Table 2:** Dietary ARA intake in mg/d (min–max) in Europe [84]

Risk group	ARA intake in mg/d (min–max)
Pregnant women	36–120 mg/d
Lactating women	90–110 mg/d
Infants (6–11 mo)	24–72 mg/d
Toddlers (1–3 y)	17 mg/d^1^
Children (4–9 y)	60–170 mg/d
Adolescents (10–18 y)	80–469 mg/d
Elderly (> 65 y)	110–317 mg/d

The values are the minimum and maximum values.^1^*n* = 1 study

*Abbreviations*: *ARA* Arachidonic acid, *mo* months of age, *y* years of age

#### Early life (infants and toddlers)

There are few data concerning dietary ARA intake in early life [3, 84]. Weaning foods contain low amounts of fat and consequently ARA [3]; potential sources are egg yolk and meat, but both are not commonly eaten by infants [90, 91]. Therefore, the dietary intake of ARA is low and much lower than that provided by both human milk and infant formulas (~ 140 mg/d) [3, 24]. For example, in the German Dortmund Nutritional and Anthropometric Longitudinally Designed (DONALD) study, the ARA intake of infants/toddlers decreased from 103 mg/d (3 mo) to 72 mg/d (6 mo) to 24 mg/d (9 mo), and the DHA intake decreased from 57 mg/d (3 mo) to 47 mg/d (6 mo) to 28 mg/d (9 mo) [90]. In these age groups, breast milk and infant formula remain the only relevant sources of ARA, with the proportion of breast milk consumed decreasing with increasing age. Low ARA intake led to significantly lower ARA plasma levels in infants who did not receive infant formula with ARA and DHA during the first six months of life [33]. This has raised concerns as to whether optimal LC-PUFA intake can or cannot be achieved through complementary foods [91]. However, the clinical effects of low ARA intake have not yet been sufficiently investigated [3].

Overall, it appears that infants will only reach the AI of 140 mg/d set by the EFSA [83] with the intake of breast milk or ARA containing infant formula [23]. As there is no AI for older children or pregnant/lactating women, no statement can be made about the adequacy of ARA intake on the basis of ARA intake alone. Nevertheless, the amount of preformed ARA provided by solid foods during late infancy and early childhood is low [3].

## Food sources of ARA

### Plant foods

Plant foods (with few exceptions, i.e., mosses [92]) contain no preformed PUFAs with a chain length of C20 or C22 [93] and thus no ARA. The precursor LA, in contrast, is abundant in oils such as safflower, sunflower, and corn oils [94]. Soy, walnut, and hemp oils, as well as walnuts, are also rich in LA [37]. Table 3 shows the contents of ALA and LA in various foods.

**Table 3:** ALA and LA contents in foods [95, 96]

Food products	ALA (n-3)	LA (n-6)	Ratio LA:ALA
g/100 g			
Plant foods			
Walnut	7.8	34.4	4.4:1
Peanut	0.5	13.9	27.8:1
Avocado	0.17	1.7	10:1
Hazelnut	0.1	8.5	85:1
Plant oils			
Linseed oil	51	16	0.3:1
Hemp seed oil^1^	16.7	57.3	3.4:1
Walnut oil	12	57	4.8:1
Rapeseed oil	9.6	22.4	2.3:1
Soybean oil	7.7	52.9	6.9:1
Wheat germ oil	7.8	55.7	7.1:1
Olive oil	0.9	8.3	9.2:1
Sunflower oil	0.5	63.1	126.2:1
Thistle oil	0.5	75.1	150.2:1

^1^Reference for hemp seed oil see [96] *Abbreviations:* *ALA* α-linolenic acid, *LA* linoleic acid

### Animal foods

ARA is found mainly in animal fats, offal, fish oils, and egg yolk [37]. Therefore, the major food groups that serve as sources for ARA in the European Union (in the following order) are eggs, pig meat, poultry meat, fish and seafood, and bovine meat (see Supplementary Table 2, Additional File 1), whereas in Australia and New Zealand, motton (goat) meat, and offal also play a role in ARA intake [89]. In 1804 adolescents (12.5–17.5 years) in the Healthy Lifestyle in Europe by Nutrition in Adolescence (HELENA) study, the main contributor to ARA intake was the food group “meat, fish, eggs, and meat alternatives” (54.2%, of which 46.2% was meat) [97]. Table 4 shows the contents of ARA and other FAs in animal source foods.

**Table 4:** Total fat and FA contents in animal source foods (g or mg/100 g) [138]

per 100 g	Total fat (g)	LA (g)	ALA (g)	ARA (mg)	EPA (mg)	DHA (mg)
Meat						
Pork (cooked)	20.1	1.0	0.3	232	33	0
Veal (cooked)	5.9	0.3	0.1	215	9	0
Lamb (cooked)	18.2	0.6	0.2	143	33	0
Beef (cooked)	9.5	0.3	0.1	43	17	0
Cold cuts (Mortadella)	29.2	1.6	0.4	62	35	39
Sausage	25.1	1.4	0.4	38	12	8
Venison						
Deer (cooked)	10.8	0.3	0.1	41	10	0
Wild boar (cooked)	8.3	0.6	0	37	15	0
Poultry						
Chicken (cooked)	9.4	2.1	113	230	7	107
Turkey (cooked)	8.7	2.2	0.1	184	124	22
Duck (cooked)	9.8	1.2	0.1	9	9	0
Fish						
Tuna (cooked)	17.3	0.3	0.3	287	1620	2435
Salmon (cooked)	12.2	1.0	0.3	63	919	1469
Trout (cooked)	3.2	0.3	0.0	30	162	577
Offal						
Kidney (veal, cooked)	7.4	0.1	0.1	113	9	0
Liver (veal, cooked)	1.4	0.2	0.0	93	4	26
Eggs and milk						
Butter	83.2	1.2	0.4	114	0	10
Fried egg	14.0	1.4	0.1	63	0	75
Hard-boiled egg	9.3	1.3	0.1	56	0	75
Scrambled eggs	12.4	3.3	0.1	49	0	64
Cheese (Gouda)	30.8	0.4	0.3	16	0	0
Milk	3.6	0.0	0.0	3	0	0
Yoghurt (3,5%)	3.8	0.1	0.1	0	0	0
Curd (40%)	11.4	0.2	0.1	0	0	0

*Abbreviations*: *ALA* α-linolenic acid, *ARA* arachidonic acid, *DHA* docosahexaenoic acid, *EPA* eicosapentaenoic acid, *LA* linoleic acid

## Results

Vegans have high intakes of grains, vegetable oils, nuts, and seeds, leading to high intakes of LA and ALA and low intakes of LC-PUFAs. As plant foods contain no preformed ARA, vegans have to rely on the endogenous synthesis of ARA from LA to meet their requirements for this LC-PUFA [93, 94]. Vegetarians also include dairy products and eggs in their diet, which slightly increases their ARA intake. One of the main research questions of this article is whether lower ARA intake is associated with lower ARA status. Therefore, studies on ARA intake and status of vegans and vegetarians are presented in Table 5 in chronological order.

**Table 5:** Arachidonic acid (ARA) intake and status in observational studies with Western adults on plant-based diets

Author (year) [Ref.], study location	Dietary assessment	Study participants (age)	Comparison of dietary ARA intake	Mean ± SD ARA intake (mg/d)	Comparison of ARA status	Mean ± SD ARA concentrations	
Studies with data on dietary ARA intake only							
Roshanai and Sanders (1984) [139], UK	7-d weighing protocol, duplicate method	24 OM, 20 VN (age not shown) 22 f, 22 m	f: OM=VN m: OM>VN (*p*<0.01)	OM: f: 100 ± 0.0^1^ m: 700 ± 300^1^ VN: 0^1^	Not assessed	Not assessed	
Beezhold et al. (2010) [140], USA	FFQ	78 OM (41.0 ± 1.4 y^2^), 60 VG (45.1 ± 1.4 y^2^) 77 f, 61 m	OM>VG (*p*<0.001)	OM: 90 ± 10^2^ VG: 10 ± 0^2^	Not assessed	Not assessed	
Rizzo et al. (2013) [141], USA and Canada	FFQ	33 634 OM, 4042 semi-VG, 6583 PES, 21 799 VG, 5694 VN (mean 59 y) f: 63.2-67.3% m: 32.7-36.8%	OM>PES>semi-VG>VG>VN	OM: 84.1 ± 0.3^3^ Semi-VG: 27.2 ± 0.7^3^ PES: 43.6 ± 0.6^3^ VG: 13.4 ± 0.3^3^ VN: 2.6 ± 0.6^3^	Not assessed	Not assessed	
Dawczynski et al. (2022) [142], Germany	5-d dietary record	65 OM, 70 FLX, 65 VG, 58 VN (18-69 y) 184 f, 74 m	OM>FLX>VG>VN (*p*<0.05)	OM: 210 (250)^6^ FLX: 110 (150)^6^ VG: 40 (40)^6^ VN: 20 (20)^6^	Not assessed	Not assessed	
García-Maldonado et al. (2023) [69], Spain	72-h dietary intake report	45 OM, 28 VG, 32 VN (25.9 ± 0.3 y^1^) 62 f, 43 m	OM>VG=VN (*p*<0.001)	OM: 160 ± 20 VG: 60 ± 20 VN: 0 ± 0	Not assessed	Not assessed	
Studies showing (partly) lower ARA status							
Fisher et al. (1986) [118], USA	no assessment	25 OM, 15 VG, 10 VN (20-47 y) 28 f, 22 m	Not assessed	Not assessed	OM>VG=VN (*p*<0.005)	PLT FAs %total: OM: 26 ± 2.5 VG: 21 ± 4 VN: 20.2 ± 5.2	
Melchert et al. (1987) [143], Germany	7-d dietary record, 24-h recall	108 OM, 102 VG, 8 VN (21-77 y) 132 f, 78 m	Not assessed	Not assessed	Serum PL: OM=VG HDL PL: OM>VG (*p* at least <0.05)	Serum PL:	
						f:	m:
						OM: 6.4 ± 1.4	6.4 ± 1.3
						VG: 6.1 ± 1.5	5.7 ± 1.4
						HDL PL:	
						f:	m:
						OM: 5.1 ± 1.0	5.0 ± 1.3
						VG: 4.4 ± 1.2	4.5 ± 1.2
Phinney et al. (1990) [93], USA	Diet history questionnaire	100 OM, 16 semi-VG, 25 VG (27.1-29.3 y) 86 f, 52 m	Not assessed	Not assessed	OM=semi-VG=VG Significantly different only for: semi-VG vs OM in PL (*p*<0.05) VG vs OM in PL and CE (*p*<0.01 and *p*=0<0.05, respectively)	FFA composition in wt%: FFA: OM: 2.4 ± 0.1^1^ Semi-VG: 2.5 ± 0.4^1^ VG: 2.0 ± 0.2^1^ TG: OM: 1.6 ± 0.1^1^ Semi-VG: 1.6 ± 0.1^1^ VG: 1.6 ± 0.1^1^ PL: OM: 12.8 ± 0.2^1^ Semi-VG: 11.5 ± 0.5^1^ VG: 11.1 ± 0.4^1^ CE: OM: 7.8 ± 0.2^1^ Semi-VG: 7.3 ± 0.5^1^ VG: 6.5 ± 0.2^1^	
Sanders and Roshanai (1992) [120], UK	7-d weighed food record and analysis of 3-d duplicate food intake	20 OM (f: 32 ± 2.2 y, m: 33 ± 1.6 y), 20 VN (f: 32 ± 2.2 y, m: 32 ± 1.6 y) 20 f, 20 m	OM>VN(*p*<0.01)	OM: f: 110 ± 30^1^ m: 130 ± 40^1^ VN: f: 0^1^ m: 0^1^	OM>VN (*p* < 0.01)	Weight % of total FA PL: OM: 27.8 ± 0.39^1^ VN: 26.2 ± 0.36^1^	
Li et al. (1999a) [108], Australia	12-d weighed diet record	24 OM (21-45y), 50 VG (21-55 y) 74 f, 0 m	Not assessed	Not assessed	Serum concentration (mg/100 ml): OM>VG (*p* = 0.004) Serum composition (% of total FA): OM=VG (p = 0.292)	Serum concentration (mg/100 ml): OM: 13.4 ± 4.0 VG: 10.5 ± 3.0 Serum composition (% of total FA): OM: 9.8 ± 1.8 VG: 9.3 ± 2.3	
Li et al (1999b) [119] Mann et al. (2006) [8], Australia	FFQ	18 HME, 60MME, 43 VG, 18 VN (20-55 y) 0 f, 139 m	HME>MME>VG>VN (*p*<0.01)	HME: 200 ± 100 MME: 100 ± 0 VG: 0 ± 0 VN: 0 ± 0	Plasma PL: HME=MME>VN=VG VG vs. HME/MME (*p*<0.05) PLT PL: HME=MME>VG>VN (*p* at least 0.05)	Plasma PL (mg/100 mL): HME: 10.6 ± 1.6 MME: 10.5 ± 1.7 VG: 9.5 ± 1.9 VN: 10.6 ± 1.5 PLT PL %total FA: HME: 24.4 ± 1.4 MME: 24.5 ± 1.2 VG: 23.9 ± 1.3 VN: 23.0 ± 1.7	
Actis et al. (2005) [144], Argentina	FFQ	10 OM, 10 VG (18-50 y) 13 f, 7 m	OM>VG (*p*=0.0001)	(% of total FAs) OM: 0.20 ± 0.08 VG: 0.02 ± 0.01	OM>VG (*p* = 0.045)	Saliva (% total FAs): OM: 4.52 ± 0.77 VG: 3.93 ± 0.46	
Kornsteiner et al. (2008) [112], Austria	24-h recall, FFQ	23 OM, 13 semi-OM, 25 VG, 37 VN (29.5-38.5y) 60 f, 38 m	OM>VG (*p*<0.05) OM>semi-OM (*p*<0.05)	OM: 205 ± 247 Semi-OM: 20 ± 17 VG: 34 ± 33 VN: 46 ± 120	SPL: OM>VG>semi-OM>VN OM vs. VN (*p*<0.05) PE: OM>VN (*p* ≤ 0.07)	mol% of total FAs: SPL: OM: 10.1 ± 1.5 Semi-OM: 9.1 ± 1.6 VG: 9.8 ± 1.4 VN: 9.0 ± 1.3 SL: OM: 3.9 ± 1.8 Semi-OM: 4.0 ± 1.5 VG: 3.6 ± 1.2 VN: 3.4 ± 1.5 PC: OM: 3.7 ± 0.9 Semi-OM: 3.0 ± 0.9 VG: 3.1 ± 0.5 VN: 3.3 ± 1.0 PS: OM: 13.1 ± 2.6 Semi-OM: 12.3 ± 3.0 VG: 13.2 ± 2.5 VN: 13.1 ± 2.9 PE: OM: 16.4 ± 2.9 Semi-OM: 15.2 ± 2.4 VG: 16.4 ± 2.5 VN: 14.7 ± 2.2	
Miles et al. (2019) [145], USA	24-h recall, FFQ	402 OM, 38 semi-VG, 104 PES, 224 VG, 72 VN (56.3-62.6y) 546 f, 294 m	Not assessed	Not assessed	OM=semi-VG OM>PES, VG, and VN (*p*<0.0001)	Adipose tissue (% of total FAs): OM: 0.5 (0.5, 0.6)^5^ Semi-VG: 0.5 (0.4, 0.6)^5^ PES: 0.4 (0.4, 0.5)^5^ VG: 0.4 (0.4, 0.5)^5^ VN: 0.4 (0.3, 0.5)^5^	
Gogga et al. (2024) [146], Poland	7-d dietary record	31 OM (35.6 ± 10.4 y), 9 PES (27.8 ± 7.7 y), 28 VG (27.2 ± 4.4 y), 30 VN (28.5 ± 6.3 y) 102 f, 0 m	Not assessed	Not assessed	OM>VN=VG (*p*<0.001) PES n. s. different from other groups	Serum: OM: 3.7 ± 0.9 PES: 3.0 ± 1.1 VG: 2.6 ± 1.0 VN: 2.5 ± 1.0	
Klein et al. (2025) [114], Germany	5-d dietary protocol	62 OM, 69 FLX, 64 VG, 57 VN (18-70 y) f: 70% m: 30%	Not assessed	Not assessed	Plasma: OM>FLX=VG=VN (*p*<0.05) RBC: OM=FLX=VG>VN (*p*<0.05)	Plasma (%FAME): OM: 6.57 (1.82)^6^ FLX: 5.43 (1.80)^6^ VG: 5.34 (1.57)^6^ VN: 5.20 (1.79)^6^ RBC (%FAME): OM: 13.98 (2.38)^6^ FLX: 13.61 (1.79)^6^ VG: 13.50 (1.46)^6^ VN: 12.93 (2.66)^6^	
Studies showing comparable or (partly) higher ARA status							
Sanders et al. (1978) [107], UK	No assessment	22 OM, 22 VN (21-66 y) 20 f, 24 m	Not assessed	Not assessed	Plasma choline phosphoglyceride: VN>OM (*p*<0.05) RBC: OM=VN	mg/g FAME: Plasma choline phosphoglycerine: OM: 91 ± 4.1^2^ VN: 106 ± 5.5^2^ RBC: OM: 125 ± 2.4^2^ VN: 126 ± 4.5^2^	
Agren et al. (1995) [111], Finland	7-d food records (household measures)	11 OM (mean 51 y), 8 with strict uncooked VN diet (mean 47 y) 17 f, 2 m	Not assessed	Not assessed	all but PS and PLT: VN=OM PS: VN>OM (*p*<0.001) PLT: OM>VN (*p*<0.001)	FA composition (mol%): total RBC: OM: 13.7 ± 1.0 VG: 14.3 ± 1.3 PE: OM: 23.8 ± 1.8 VG: 24.1 ± 2.1 PC: OM: 5.8 ± 1.2 VG: 7.5 ± 3.0 PS: OM: 23.1 ± 1.7 VG: 28.5 ± 2.4 CE: OM: 5.1 ± 1.0 VG: 5.0 ± 1.2 TG: OM: 1.3 ± 0.4 VG: 1.4 ± 0.5 FFA: OM: 0.6 ± 0.3 VG: 0.6 ± 0.3	
Fokkema et al. (2000) [94], Netherlands	FFQ	15 OM, 12 VN (20-60 y) 10 f, 17 m	Not assessed	Not assessed	OM=VN	composition (mol%): RBC: OM: 13.8 ± 1.2 VN: 14.2 ± 1.1 PLT: OM: 19.8 ± 2.6 VN: 19.3 ± 3.2 CE: OM: 6.8 ± 1.6 VN: 6.2 ± 1.5 TG: OM: 1.5 ± 0.6 VN: 1.4 ± 0.3	
Rosell et al. (2005) [147], United Kingdom	FFQ	196 OM, 231 VG, 232 VN (20-78 y) 0 f, 659 m	Not assessed	Not assessed	OM=VG=VN	total FAs: OM: 1.2 (1.1, 1.4)^5^ VG: 1.0 (1.0, 1.1)^5^ VN: 1.1 (1.0, 1.2)^5^	
Sarter et al. (2015) [116], USA	3 d 24-h Recall	78 OM, 40 VN (20-54 y) (sex distribution of subsample not shown)	Not assessed	Not assessed	OM=VN	% of total FAs: OM: 12.9 ± 1.7 VN: 12.0 ± 1.9	
Elorinne et al. (2016) [148], Finland	3-d food record, questionnaire	19 OM, 22 VN (24-52 y) 27 f, 14 m	Not assessed	Not assessed	OM=VN	% of total FAs: OM: 6.9 ± 1.1 VN: 6.3 ± 1.4	
Pinto et al. (2017) [113], UK	FFQ	24 OM, 23 VN (40-70 y) 27 f, 20 m	Not assessed	Not assessed	Plasma, RBC: OM=VN	weight% FAs: Plasma: OM: 6.7 (6.1, 7.3)^5^ VN: 6.6 (5.9, 7.2)^5^ RBC: OM: 15.9 (14.9, 16.9)^5^ VN: 15.6 (14.4, 16.9)^5^	
Chamorro et al. (2020) [9], Chile	24-h recall, FFQ	33 OM (without marine foods), 34 VN (18-25 y) 0 f, 67 m	OM>VN (no *p* value, because no intake in vegan group)	OM: 343 ± 76 VN: 0	Plasma: OM>VN (*p*=0.003) RBC and spermatozoa: OM=VN	Plasma (mol% of FA): OM: 6.3 ± 1.6 VN: 4.8 ± 1.5 RBC (g/mol% of FA): OM: 11.9 ± 2.8 VN: 10.6 ± 1.8 Spermatozoa (g/mol% of FA): OM: 2.0 ± 1.0 VN: 1.6 ± 1.1	
Craddock et al. (2022) [115], Australia	App-based 7-d food and training diary	8 OM, 12 VN (18-55 y) 0 f, 20 m	Not assessed	Not assessed	OM=VN	Whole blood: OM: 9.4 (8.2, 10.7)^5^ VN: 8.6 (7.9, 9.3)^5^	
Menzel et al. (2022) [122], Germany	3-day weighed food records	36 OM (38.5 [32.0-46.0] y), 36 VN (37.5 [32.5-44.0] y) 36 f, 36 m	OM>VN (*p*<0.0001)	OM: 166 (115-240)^6^ VN: 5.5 (3.6-11)^6^	OM=VN	Plasma PL (%): OM: 8.8 (8.2-9.4)^7^ VN: 8.2 (7.7-8.8)^7^	
Chamorro et al. (2023) [1], Chile	FFQ	35 OM (21.9 ± 2.8 y), 34 no-fish-OM (23.5 ± 2.7 y), 36 PES (24. ± 2.4 y), 35 VN (23.0 ± 3.3 y) 0 f, 140 m	no-fish-OM=OM>PES>VN (*p*=0.002)	OM: 343 ± 59 no-fish-OM: 385 ± 76 PES: 155 ± 39 VN: 25 ± 5	Plasma: OM=no-fish-OM=PES>VN (*p* = 0.023) RBC: OM=no-fish-OM=PES=VN Spermatozoa: OM=no-fish-OM=PES=VN	Plasma (mol% of FA): OM: 6.5 ± 1.8 no-fish-OM: 6.4 ± 1.0 PES: 6.3 ± 0.8 VN: 4.2 ± 0.3 RBC (g/mol % of FA): OM: 11.2 ± 2.1 no-fish-OM: 11.9 ± 2.5 PES: 10.8 ± 1.5 VN: 10.3 ± 1.0 Spermatozoa (g/mol% of FA): OM: 2.2 ± 0.6 no-fish-OM: 2.2 ± 0.3 PES: 2.1 ± 0.4 VN: 2.3 ± 0.6	
Groufh-Jacobsen et al. (2025) [117], Norway	Dietary screener, 24-h dietary recalls	71 OM, 25 FLX, 30 PES, 20 VG, 19 VN (16-24 months) 125 f, 40 m	Not shown	Not shown	OM=FLX=VG=VN OM>PES (*p*=0.003)	Whole blood (%FAME): OM: 12.0 ± 1.7 PES: 10.5 ± 1.8 FLX: 11.4 ± 1.7 VG: 11.1 ± 1.3 VN: 11.4 ± 1.8	

The values without superscripts are presented as the means ± SDs.^1m^ean ± SEM; ^2^mean ± SE; ^3^mean ± SE (standardized to 2000 kcal/day); ^4^intake % of total FAs; ^5^mean (95% CI); ^6^median (IQR); ^7^geometric means (95%-CI)

*Abbreviations*: *CE* cholesteryl esters, *En%* energy%, *f* female, *FAs* fatty acids, *FAME* FA methyl esters, *FFA* free FAs, *FFQ* food frequency questionnaire, *FLX* flexitarian, *HDL* high-density lipoprotein, *HME* high meat eaters, *m* male, *MME* moderate meat eaters, *OM* omnivore, *PC* phosphatidyl-choline, *PE* erythrocyte phosphatidylethanolamine, *pescatarians* pescatarians, *PL* phospholipids, *PLT* platelets, *PS* phosphatidyl serine, *RBC* red blood cells, *semi-VG* semivegetarians, *SL* sphingolipids, *SPL* erythrocyte spingho- and phospholipids, *TG* triglycerides, *VG* vegetarians, *VN* vegans

### ARA intake in vegetarians and vegans

Of all included studies with adults, five studies reported on ARA intake only. A further seven studies reported on both intake and status of ARA in vegetarians/vegans (Table 5). Taken together, the studies confirmed a lower ARA intake in vegetarian and vegan adults than in omnivorous adults. The average daily ARA intake was usually between 0–60 mg/d for vegetarians, 0–46 mg/d for vegans, and 84–700 mg/d for omnivores.

### ARA status in vegetarians and vegans

The results regarding the ARA status are classified into studies that show a (partially, i.e., only in subfractions of PL) lower, a comparable or a (partly) higher ARA status in vegetarians/vegans. If there were differences in plasma/serum PL but not RBC, this was classified as comparable because of the better informative value of RBC in the long term (see discussion). Direct comparison of the study results is difficult because methodological differences in FA analysis, i.e., relative FA patterns, depend on the amount of FA that can be quantified by the chosen method. The analysis of absolute FA concentrations is independent of this. Thus, some authors suggest that quantifying absolute FA concentrations is advantageous because they can be evaluated independently from one another [56]. As the studies showed results in relative *and* absolute FA concentrations, we have therefore reported the percentage deviation within a study for the studies that showed (partly) lower ARA status in Table 5. If there was a difference between omnivores and vegetarians/vegans ARA status, in approximately 60% of the studies (12 of 20 comparisons), vegetarians/vegans had ARA concentrations between 2 and 15% lower than those of omnivores.

In addition to the in Table 5 presented observational studies, three interventional studies from Norway, Australia, and the U.S. investigated the effects of switching from an omnivorous to a vegetarian or vegan diet on ARA status, with the result that ARA intake [74, 98] decreased markedly. After two weeks of a very low-fat vegetarian diet, there were no differences in the ARA PL fraction but significantly increased ARA concentrations in the serum CE [98]. In a single-blind RCT, ARA plasma PL levels in patients with RA declined after a 3.5-month duration of a vegan diet but recovered after a 9-month duration of a vegetarian diet [99].

### ARA intake and status in pregnant women, breastfeeding women, children, and adolescents

In general, a mother’s diet has a strong influence on fetal and human milk lipids [100–102]. However, as mentioned above, ARA transfer via the placenta is not associated with maternal diet or ARA status, and the ARA concentration in breast milk is relatively stable at approximately 0.5% milk FAs [24]. This is confirmed by a review showing that breast milk samples from women living in low- and middle-income countries, where the typical food is mainly plant-based, do not have considerably lower ARA levels than women from high-income countries with a higher intake of animal foods [103]. Nevertheless, in vulnerable groups of pregnant and lactating women and their offspring, it is important to know whether there are differences between vegetarian, vegan, and omnivorous subjects. Therefore, studies involving pregnant and breastfeeding women as well as infants and children are presented in Table 6. There are studies in which pregnant Asian women had higher ARA blood levels in the vegetarians than in the omnivores [104–106]. However, owing to genetic differences in the haplotypes between Asian and European populations [35, 39, 40], these studies were not included in our analysis. Nevertheless, the authors also reported no differences in ARA concentrations in the cord plasma and breast milk of vegetarians vs. omnivorous women [104, 105] or even higher ARA concentrations in arterial PL or cord plasma in vegetarian women than in omnivorous women [104, 106].

**Table 6:** Arachidonic acid (ARA) intake and status in observational studies with risk groups on plant-based diets in Western countries

Author, year [Ref.], study location	Dietary assessment	Study participants (age)	Comparison of dietary ARA intake	Median (IQR) ARA intake (mg/d)	Comparison of ARA status	ARA concentrations
Studies with pregnant women						
Lakin et al. (1998) [110], UK	FFQ in late pregnancy (38-42th weeks of gestation)	10 OM (28 ± 6 y), 4 VG (27 ± 3 y)	OM > VG (*p* < 0.01)	OM: 198 ± 51^1^ VG: 42 ± 17^1^	RBC, placenta, cord: OM = VG	Mean ± SD (wt%): RBC: OM: 11.9 ± 3.4 VG: 14.3 ± 1.2 Placenta: OM: 22.5 ± 0.8 21.5 ± 0.9 Cord: OM: 8.4 ± 3.8 VG: 7.9 ± 2.8
Crozier et al. (2019) [2], UK	FFQ in early and/or late pregnancy	169 VG, 4693 OM (20–34 y)	Not shown	Not shown	Early pregnancy: VG < OM (*p* = 0.02), Late pregnancy: VG < OM (*p* = 0.03)	Median (IQR) (µg/mL): Early pregnancy: OM: 165.8 (135.5, 200.8) VG: 143.9 (134.8, 170.1) Late pregnancy: OM: 108.4 (83.8, 139) VG: 100.9 (72.9, 122.7)
Koeder et al., PREGGIE study (unpublished data), Germany	3-d weighed dietary record, t1: 9‒16th; t2: 35‒38th week of gestation	13 OM, 32 VN plus newborns (18–39 y)	t1 and t2: OM > VN (*p* < 0.001)	t1: OM: 121 (57.9–365.7) VN: 5.1 (2–14.3) t2: OM: 138.4 (61.3–204.7) VN: 6.4 (2.8–19.4)	t1: OM > VN (*p* < 0.05) t2: OM = VN	Mean ± SEM (mg/l): t1: OM: 369.1 ± 33.9 VN: 273.2 ± 16.1 t2: OM: 335.7 ± 34.3 VN: 269.2 ± 18.9
Studies with breastfeeding women (and their infants)						
Sanders et al. (1978) [107], UK	No assessment	Breast milk of 4 OM and 4 VN (age not shown) RBC of exclusively breastfed infants of 3 VN and 6 OM mothers	No assessment	No assessment	Breastmilk: OM = VN RBC infants: OM = VN	Mean ± SE (%FAME): Breast milk: OM: 5.4 ± 1.3 VN: 7.2 ± 2.1 RBC FA infants: OM: 137 ± 6.2 VN: 133 ± 6.1
Finley et al. (1985) [149], USA	24-h dietary recall (t0), monthly 2-d dietary records	29 OM, 30 VG, 8 Semi-VG/fish-eaters with 172–242 breast milk samples (22–37 y)	Not shown	Not shown	: OM = VG	Mean ± SD (% in milk fat): OM: 0.3 ± 0.1 VG: 0.3 ± 0.1
Specker et al. (1987) [150], USA	3-day-diet diaries from 16 of the women	Breast milk of 7 OM, 12 VN on macrobiotic diet^2^ (22–35 y)	Not shown	Not shown	OM = VN	Mean ± SEM (%): OM: 0.5 ± 0.0 VN : 0.7 ± 0.0
Perrin et al. (2019) [134], USA	Digital questionnaire	Breastmilk of 26 OM (31.0 ± 4.7 y), 22 VG (32.2 ± 4.6 y), 26 VN (32.7 ± 5.2 y)	Not shown	Not shown	OM = VG = VN	Mean and IQR (g/dl): OM: 0.45 (0.16) VG: 0.38 (0.13) VN: 0.38 (0.21)
Studies with toddlers and children						
Krajcovicova-Kudlackova et al. (1997) [136], Slovakia	Dietetic questionnaire	19 OM, 10 Semi-VG, 15 VG, 7 VN (11–15 y) 27 f, 24 m	Not shown	Not shown	OM = Semi-VG = VG = VN	Mean ± SD (% of total FA): OM: 7.5 ± 0.2 Semi-VG: 7.1 ± 0.2 VG: 7.1 ± 0.2 VN: 6.9 ± 0.3
Weder et al. (2022) [151], Germany	3-d weighed dietary record	164 OM, 127 VG, 139 VN (1–3 y) 223 f, 207 m	OM > VG > VN (*p* < 0.0001)	VN: 7.2 (2.6–16.3) VG: 12 (5.4–21.7) OM: 34.3 (21.9–54.6)	No assessment	No assessment

^1^ Mean ± SD. ^2^ restrictive diet witout meat, eggs, and dairy products

*Abbreviations*: *En%* energy%, *f* female, *FA* fatty acids, *%FAME* % of total fatty acids methyl esters, *FFQ* food frequency questionary, *FLX* flexitarian, *m* male, *OM* omnivore, *RBC* red blood cells, *VG* vegetarians, *VN* vegans

## Discussion

### ARA intake and status in adults

Despite the definitive lower dietary ARA intake of vegans and vegetarians, investigators have reported different results concerning the influence of these plant-based diets on the levels of ARA and its intermediates, elongation, and desaturation products compared with omnivores. Most studies showed (partially) lower (*n* = 11) or comparable (*n* = 12) ARA concentrations in subjects with plant-based diets than in those with omnivorous diets. Only one study reported partly higher ARA concentrations in vegans [107]. Thus, as Burns-Whitmore et al. stated in their review from 2019 with 13 studies, “there are inconsistent findings of A(R)A tissue concentrations compared to omnivores” [5].

On the one hand, one could assume that lower LC-PUFA concentrations in some subfractions of PL in eleven studies may show that the dietary intake of preformed LC-PUFAs is more effective than in vivo synthesis from LA (and ALA) [108]. On the other hand, while the FA concentration in plasma/serum PL is more influenced by diet in the short term/by the most recent meal, RBC reflects habitual FA intake (approximately 120 days [109]) [108, 110]. In studies focusing on FA status in RBC [1, 9, 94, 107, 111–114] or whole blood [115–117], ARA status was not significantly different (except for ARA in SPL and PE in [112] and in [114]). This was true for both the vegans and the vegetarians. Nevertheless, a longer duration is also needed for the diet to influence platelet PL FA concentrations [109]. Four studies reported higher ARA rates in platelets from omnivorous participants than in those from vegan/vegetarian participants [111, 118–120], and one study reported no differences between omnivores and vegans on average [94]. However, there were only minor increases in platelet ARA levels (< 10% difference) of high meat eaters to vegans and no differences in template bleeding times or platelet aggregation [118–120]. The comparable platelet aggregation suggests that even the lower ARA concentration in platelets is sufficient to supply normal aggregation [118] and, therefore, does not imply an ARA status that could be classified as too low.

Overall, as the RBC ARA concentration, a long-term marker for adequate ARA status, mostly did not differ between meat-eaters and plant-based eaters, it appears that the endogenous conversion of LA in vegetarians and vegans is sufficient for ARA synthesis. In addition, the mentioned intervention studies revealed no differences in ARA concentrations or recovery of ARA concentrations after some time when omnivores were switched to a vegan diet, confirming this assumption. This may be due to adaptations in ARA biosynthesis, as ALA can also be converted to EPA and DHA in long-term plant-based diets [5, 116]. Furthermore, although ARA differences in tissues other than RBC between the groups are present, the relative differences were moderate (mainly ≤ 15%). In comparison, other FAs, such as DHA and EPA, vary much more because of differences in intake [121]. It can also be argued that omnivores tend to have too high an ARA level, possibly favoring chronic inflammatory diseases, and that lower ARA levels are, on the contrary, advantageous [39]. The Nutritional Evaluation (NuEva) study analyzed 703 fatty acid profiles in plasma and RBCs lipids across omnivores (Western diet; *n* = 62), flexitarians (*n* = 69), vegetarians (*n* = 64), and vegans (*n* = 57). n-6 FA concentrations, particularly LA, were highest in vegans and vegetarians. Conversely, omnivores had higher ARA in plasma and RBCs. Vegans had lower n-3 FAs in both plasma and RBCs, also reflected in a lower n-3 index (EPA + DHA) values (*p* < 0.05), indicating a trend with restriction of animal foods: omnivores/flexitarians > vegetarians > vegans. IL-6, IL-8, IL-10, TNF-α and high-sensitive C-reactive protein (hsCRP) did not differ between groups, and vegans had lower leptin levels compared to omnivores. As expected, the latter is directly related to the body fat percentage. Thus, our data indicate that the lower plasma ARA concentrations in vegetarians and vegans (as well as the lower ARA levels in RBC lipids in vegans) have no influence on the inflammatory status in a healthy collective [114]. Menzel et al. [122, 123] described comparable findings.

When evaluating these results, however, it must be noted that the FA desaturase enzymes genotype was not reported in the included studies. However, as we chose to include only studies with participants from Western societies, we assume that the distribution of genotypes within the populations should be comparable between the investigated groups.

With respect to n-3 FAs, it is evident that the majority of vegans and vegetarians have a lower dietary intake and status of EPA and DHA. However, considering the well-documented lower cardiovascular risk factors in subjects on plant-based diets, there is currently a lack of evidence for additional heart health benefits by increasing the EPA/DHA intake in vegetarians and vegans. Nonetheless, there are other health-related concerns related to low DHA intake, such as cognitive dysfunction, depression, a neurologic decline later in life or a greater risk for suicidal ideation [116, 124]. The authors of a recent scoping review of systematic reviews of prospective studies revealed, however, that “pre- and postnatal polyunsaturated fatty acids (PUFAs) intake was not consistently associated with growth, neurological, visual and cognitive outcomes, allergic diseases, cardiovascular, and metabolic health in childhood” [125]. The DHA/EPA and ARA levels interact with each other. Consequently, if individuals supplement with EPA/DHA, it may be advisable not to overdose them to prevent a decrease in ARA concentrations, i.e., to take EPA/DHA supplements only up to 250–500 mg/d. As mentioned above, it seems important to balance the intake of ARA and EPA/DHA [10, 73].

### ARA intake and status in pregnant women, breastfeeding women, children, and adolescents

Very few studies have investigated ARA intake and ARA status of pregnant (*n* = 3) and breastfeeding (*n* = 4) women. While one of the three studies involving pregnant women did not reveal differences in ARA levels, the other two studies reported lower ARA concentrations in pregnant women with vegetarians and vegans. One explanation for the lower ARA levels reported in the study by Crozier et al. (2019) is that the lower n-3 PUFA status in their study led to the conversion of ARA to DPAn-6, whose concentration was greater in vegetarian women than in omnivorous women [2]. This could indicate that ARA was present in sufficient quantities in vegetarian subjects and had therefore been converted into DPAn-6 as a storage form.

The results of the PREGGIE study with vegan and omnivorous pregnant women revealed that the ARA status did not differ significantly at t2, indicating that ARA levels might be maintained during pregnancy (Koeder et al., unpublished data). Overall, there are too few data concerning ARA status in pregnant women. However, because ARA concentrations were not statistically different, together with the studies with not pregnant women/men, it seems likely that there is no need to supplement ARA in pregnancy on a vegan or vegetarian diet. It should also be noted that ARA and DHA are preferentially transported in the placenta probably due to the special physiological importance of these FAs for fetal development [126–128]. In contrast, ARA supplementation combined with low n-3 LC-PUFA status could promote inflammatory conditions associated with the development of common neurodevelopmental disorders in infants [129]. Furthermore, vegetarianism during pregnancy was not associated with poorer neurocognitive development in children aged 6–7 years in the study by Crozier et al. (2019). However, the authors noted that the statistical power might be too low to detect clinically relevant differences between the groups.

None of the included studies showed differences in ARA concentrations in human milk between vegan/vegetarian and omnivorous women. This finding aligns well with the suggestion of most experts that ARA concentrations are relatively stable and not influenced by maternal diet [15, 23–27]. The dose-dependent increase in ARA in breastmilk shown in one RCT [28] was also in the range where ARA concentrations are relatively stable according to Brenna et al. [24]. This could indicate that a minimum level of ARA is maintained. Furthermore, there were no significant differences in plasma ARA concentrations in infants breastfed by vegetarian/vegan mothers. Therefore, it seems unnecessary for lactating women to supplement ARA. One could argue that the mother's health might suffer from the depletion of ARA, which is passed on to the infant through breast milk. However, it is likely that the body’s own stores are filled by the internal synthesis of ARA from LA. For infants up to the age of 6 months, ARA is necessary and recommended, either through breast milk or infant formula. Researchers have not yet determined whether preformed ARA is needed with the introduction of weaning food that is low in ARA. The decrease in ARA concentrations after birth may demonstrate the likely insufficient biosynthetic capability to meet the infant’s demand [31, 34]. In contrast, it could also be hypothesized that the infants’ requirements are lower after birth and that ARA synthesis is therefore downregulated.

Only two studies investigated the ARA intake and/or status of vegetarian and vegan children. Although no differences were found in the ARA status between omnivorous, vegetarian, semi-vegetarian, and vegan children in Slovakia (*n* = 51), no definitive conclusions can be drawn from only one study (the other study reported no ARA status). However, in nonvegetarian 1- and 2-year-old toddlers in Canada, there were no associations between (LA and) ARA intake and (LA and) ARA blood status (plasma total PL, RBC phosphatidylethanolamine and phosphatidylcholine) [91], which is in line with the findings of a previous study in young children (1–11 y) in the US [130]. Wiedeman et al. hypothesized that their findings “may be explained, in part, by mechanisms regulating ARA composition and use and suggest that circulating ARA amounts are tightly regulated during development” [91]. As a result, ARA supplementation in toddlers and older children on vegetarian or vegan diets may not be necessary.

Overall, there is a need for further research on the effects of plant-based diets on ARA levels, especially in pregnant and lactating women, infants, children, and adolescents. Particular attention should be given to the interactions of ARA with DHA (+ EPA). Regarding all diets, there is also a need for further research on ARA requirements in infants and young children and on the point at which the body's own synthesis of ARA is sufficient to cover its own requirements.

## Conclusion and preliminary recommendations for the dietary intake of ARA

### General recommendations for healthy adults in Western countries

There is no generally recognized reference range for simply determining whether an individual’s ARA level is physiological. However, for healthy adults on a vegetarian or vegan diet, we do not recommend ARA supplementation, as the internal conversion of LA to ARA seems to be sufficient. Even slow converters most likely are able to synthesize enough ARA because of the higher LA intake in plant-based diets. To be on the safe side, the dietary and metabolic factors that inhibit the conversion of LA to ARA, such as saturated fats, cholesterol, trans-FAs, and alcohol [131], should be limited. For optimal LA conversion to ARA, it might be advisable to maintain an n-6 (LA):n-3 (ALA) ratio > 1:1 because otherwise, n-3 FAs are preferentially converted, compared to n-6 FAs [132], which consequently might lead to lower ARA levels. However, in most cases, the LA:ALA ratio of vegetarians (13:1), especially vegans (19:1), is much higher than that of omnivores (9:1) [8]. Davis and Kris-Etherton (2003) suggested an n-6:n-3 ratio of 2:1–4:1 for vegetarians/vegans and those who do not receive preformed EPA or DHA. Again, this recommendation refers to healthy adults, with possible exceptions for specific subgroups (e.g., patients with inflammatory diseases).

### Additional recommendations for pregnant women

There are too few data to derive final recommendations for vegetarian and vegan pregnant women. Nevertheless, there seems to be no urgent need for ARA supplementation, as ARA transfer via the placenta does not seem to be related to the mother’s ARA status and intake [23]. The depletion of maternal ARA stores is also likely prevented because the stores are replenished by the internal synthesis of ARA from LA.

### Additional recommendations for lactating women

Given that the ARA concentration in breast milk is relatively stable and that there are no significant differences in ARA levels in the milk of vegetarian and vegan women compared with that of omnivorous women, we do not recommend ARA supplementation in lactating women. Moreover, we recommend breastfeeding in general for at least 4‒6 months and then partially breastfeeding with two breast milk meals in addition to weaning foods until the first birthday. In a vegan diet, breast milk offers many benefits even after the child’s first birthday. One study revealed that, after 12 months of breastfeeding, ARA and DHA concentrations even increased in comparison to those in early milk (< 12 months) [133]. However, the depletion of maternal ARA stores is likely prevented because the stores are replenished by the internal synthesis of ARA from LA.

### Infants (0–12 months)

There are too few data to derive final recommendations for vegetarian and vegan infants. Owing to insufficient ARA self-synthesis because of the limited conversion of LA to ARA, infants need preformed ARA, either via breast milk or via infant formula [3, 32]. As a result, in 2020, the European Academy of Pediatrics and the Child Health Foundation published a position paper stating that infant formula should provide DHA alongside ARA, as infant formula without ARA may not be suitable or safe for healthy infants [23]. This is true for all types of diets. Studies with breastfed infants indicate sufficient amounts of ARA from breast milk for lactating women on a vegetarian or vegan diet [107, 134]. If breastfeeding is not possible, infant formula with ARA is urgently recommended in the first year of life. Here, the EFSA recommends 140 mg/d ARA in the first six months [83] and a balanced intake of ARA:DHA of at least 1:1 should be maintained [18, 135]. As weaning foods usually contain low amounts of ARA [3], the intake of ARA should perhaps be increased. However, there are no recommendations for ARA intake beyond 6 months of age in Europe. Moreover, lower ARA intake at this development stage could lead to physiological ARA status and may not have clinical consequences.

### Toddlers (1–3 years)

Because ARA accumulates in body tissues in the first two years of life [3, 18], it seems particularly important to meet the ARA demand during this time. There is only one study on the ARA status of vegan and vegetarian toddlers. Therefore, no final recommendations for toddlers can be derived. Since ARA intake may be too low in young children, there may be a need to set appropriate estimated average requirements, especially for this age group [91]. However, the two above mentioned studies with toddlers (1- and 2-year-old) and young omnivorous children (1–11 y) revealed no relations between ARA intake and blood concentrations, indicating that the ARA status is tightly regulated and that endogenous ARA biosynthesis covers the need for ARA during development [91, 130]. Thus, ARA supplementation in toddlers on vegetarian or vegan diets is not recommended.

### Children and adolescents

There was one study with children/adolescents aged 11–15 years in Slovakia, where the authors reported no differences in ARA status among the omnivores, semi-vegetarians, vegetarians, and vegans [136]. This indicates, together with the data from Orton et al. [130] and Wiedeman et al. [91], that ARA biosynthesis is sufficient at this age.

## Closing remarks

According to the available data, it can be assumed that adults, including pregnant and breastfeeding women, as well as infants, children, and adolescents, are able to produce sufficient amounts of ARA via plant-based diets. Therefore, ARA supplementation in vegetarian and vegan diets is apparently not necessary in Western populations. However, very little data are available on the risk groups mentioned. Moreover, owing to the lack of well-established reference ranges, only relative differences in ARA status between vegetarians/vegans and omnivores can be determined, and no deficiency can be diagnosed. All in all, these recommendations should be regarded as preliminary owing to insufficient data, and further studies with risk groups on vegetarian and vegan diets are urgently needed.

## Supplementary Information

Additional file 1: Table S1. Nutritionally important n-6 and n-3 PUFA. Table S2. Median per capita daily intake of ARA from food sources in different regions and countries.

## Data Availability

No datasets were generated or analysed during the current study.

## Abbreviations

AHS: Adventist-Healthy-Study
AI: Adequate intake
ALA: α-Linolenic acid
ARA: Arachidonic acid
CE: Cholesteryl esters
CHD: Congenital heart disease
CVD: Cardiovascular disease
DGE: Deutsche Gesellschaft für Ernährung, German Nutrition Society
DGLA: Dihomo-γ-linolenic acid
DHA: Docosahexaenoic acid
DONALD study: Dortmund Nutritional and Anthropometric Longitudinally Designed Study
DPA: Docosapentaenoic acid
DRV: Dietary reference value
DTA: Docosatetraenoic acid (adrenic acid)
E%: % total energy
EDA: Eicosadienic acid
EFSA: European Food Safety Authority
EPA: Eicosapentaenoic acid
EPIC: European Prospective Investigation into Cancer and Nutrition
FA: Fatty acid
FAO: Food and Agriculture Organization of the United Nations
FFA: Free fatty acid
FLX: Flexitarian
GLA: γ-Linolenic acid
HDL: High-density lipoprotein
HELENA study: Healthy Lifestyle in Europe by Nutrition in Adolescence Study
HME: High-meat eaters
hsCRP: High-sensitive C-reactive protein
IL: Interleukin
LA: Linoleic acid
LC-PUFAs: Long-chain polyunsaturated fatty acids
LTB_4_: Leukotrien B_4_
MME: Moderate-meat eaters
NDA: Dietetic Products, Nutrition and Allergies
NuEva study: Nutritional Evaluation Study
PGE_2_: Prostaglandin E_2_
PL: Phospholipids
PS: Phosphatidylserine
PUFA: Polyunsaturated fatty acid
RA: Rheumatoid arthritis
RBC: Red blood cell
RCT: Randomized controlled trial
SD: Standard deviation
SPL: Sphingo- and phospholipids
TFAs: Trans-fatty acids
TG: Triglycerides
TNF: Tumor necrosis factor
UL: Tolerable upper intake level
WHO: World Health Organization
