# Supplementary material for: Arachidonic acid as a potentially critical nutrient for vegetarians and vegans – position paper of the Research Institute for Plant-based Nutrition (IFPE)
Source: Lipids Health Dis. 2025 Jul 19;24:244. doi: 10.1186/s12944-025-02645-z (PMC12275400; doi:10.1186/s12944-025-02645-z)
Supplement: Supplementary file 1 — Additional file 1: Table S1. Nutritionally important n-6 and n-3 PUFA. Table S2. Median per capita daily intake of ARA from food sources in different regions and countries. [file 12944_2025_2645_MOESM1_ESM.pdf]

Arachidonic acid as a potentially critical nutrient for vegetarians and vegans – position paper of the Research Institute for Plant-Based Nutrition (IFPE)

Table S1: Nutritionally important n-6 and n-3 PUFA [86]

Common name	Systematic name	N minus abbreviation	Typical sources
**n-6 PUFA**			
linoleic acid	*cis*-9, *cis*-12-octadecadienoic	18:2n-6 (LA)	most vegetable oils
γ-linolenic acid	*cis*-6, *cis*-9, *cis*-12-octadecatrienoic acid	18:3n-6 (GLA)	evening primrose, borage and blackcurrant seed oils
dihomo-γ-linolenic acid	*cis*-8, *cis*-11, *cis*-14-eicosatrienoic acid	20:3n-6 (DHGLA)	very minor component in animal tissues
arachidonic acid	*cis*-5, *cis*-8, *cis*-11, *cis*-14-eicosatetraenoic acid	20:4n-6 (AA)	animal fats, liver, egg, fish
docosatetraenoic acid	*cis*-7, *cis*-10, *cis*-13, *cis*-docosatetraenoic acid	22:4n-6	very minor component in animal tissues
docosapentaenoic acid	*cis*-4, *cis*-7, *cis*-10, *cis*-13, *cis*-16 docosapentaenoic acid	22:5n-6	very minor component in animal tissues
**n-3 PUFA**			
α-linolenic	*cis*-9, *cis*-12, *cis*-15-octadecatrienoic acid	18:3n-3 (ALA)	flaxseed oil, perilla oil, canola oil, soybean oil
stearidonic acid	*cis*-6, *cis*-9, *cis*-12, *cis*-15-octadecatetraenoic acid	18:4n-3 (SDA)	fish oils, genetically enhanced soybean oil, blackcurrant seed oil, hemp oil
	cis-8, *cis*-11, *cis*-14, *cis*-17-eicosatetraenoic acid	20:4n-3	very minor component in animal tissues

eicosapentaenoic acid	*cis*-5, *cis*-8, *cis*-11, *cis*-14, *cis*-17-eicosapentaenoic acid	20:5n-3 (EPA)	fish, especially oily fish (salmon, herring, anchovy, smelt and mackerel)
docosapentaenoic acid	*cis*-7, *cis*-10, *cis*-13, *cis*-16, *cis*-19-docosapentaenoic acid	22:5n-3 (n-3 DPA)	fish, especially oily fish (salmon, herring, anchovy, smelt and mackerel)
docosahexaenoic acid	*cis*-4, *cis*-7, *cis*-10, *cis*-13, *cis*-16, *cis*-19-docosahexaenoic acid	22:6n-3 (DHA)	fish, especially oily fish (salmon, herring, anchovy, smelt and mackerel)

Table S2: Median per capita daily intake of ARA from food sources in different regions and countries [89]

	**Average energy intake (kcal/d)**	**Per capita daily intake of ARA from food sources, mg/day**										**ARA (% of total energy intake)**
Regions/countries		Eggs	Pig, meat	Poultry, meat	Fish, seafood	Bovine, meat	Milk	Offals, edible	Mutton (goat), meat	Meat, other	Total	
European Union	3420	53.1	51.0	34.6	25.0	20.2	12.0	9.2	2.5	2.4	210	0.055
Australian and New Zealand	3170	34.2	30.5	60.6	21.4	41.3	8.7	20.4	29.9	3.0	250	0.074
USA and Canada	3650	54.5	37.6	71.4	18.5	40.2	11.6	2.7	1.4	0.7	239	0.061
China	2970	89.3	52.7	22.2	38.8	6.0	1.7	11.6	5.7	1.7	230	0.069
Japan	2810	92.3	29.9	31.4	65.0	11.0	3.9	7.9	0.4	0.2	242	0.078
Low-income countries	2643	20.5	6.8	21.2	12.0	8.2	3.1	6.6	2.9	0.7	82	0.029
